# Spatial–temporal modelling of fMRI data through spatially regularized mixture of hidden process models

**DOI:** 10.1016/j.neuroimage.2013.09.003

**Published:** 2014-01-01

**Authors:** Yuan Shen, Stephen D. Mayhew, Zoe Kourtzi, Peter Tiňo

**Affiliations:** a School of Computer Science, The University of Birmingham, Birmingham, UK; b School of Psychology, The University of Birmingham, Birmingham, UK; c Laboratory for Neuro- and Psychophysiology, K.U. Leuven, Belgium

**Keywords:** ROI-based fMRI analysis, Generative fMRI models, Clustering fMRI time series

## Abstract

Previous work investigated a range of spatio-temporal constraints for fMRI data analysis to provide robust detection of neural activation. We present a mixture-based method for the spatio-temporal modelling of fMRI data. This approach assumes that fMRI time series are generated by a probabilistic superposition of a small set of spatio-temporal prototypes (mixture components). Each prototype comprises a temporal model that explains fMRI signals on a single voxel and the model's “region of influence” through a spatial prior over the voxel space. As the key ingredient of our temporal model, the Hidden Process Model (HPM) framework proposed in Hutchinson et al. (2009) is adopted to infer the overlapping cognitive processes triggered by stimuli. Unlike the original HPM framework, we use a parametric model of Haemodynamic Response Function (HRF) so that biological constraints are naturally incorporated in the HRF estimation. The spatial priors are defined in terms of a parameterised distribution. Thus, the total number of parameters in the model does not depend on the number of voxels. The resulting model provides a conceptually principled and computationally efficient approach to identify spatio-temporal patterns of neural activation from fMRI data, in contrast to most conventional approaches in the literature focusing on the detection of spatial patterns. We first verify the proposed model in a controlled experimental setting using synthetic data. The model is further validated on real fMRI data obtained from a rapid event-related visual recognition experiment (Mayhew et al., 2012). Our model enables us to evaluate in a principled manner the variability of neural activations within individual regions of interest (ROIs). The results strongly suggest that, compared with occipitotemporal regions, the frontal ones are less homogeneous, requiring two HPM prototypes per region. Despite the rapid event-related experimental design, the model is capable of disentangling the perceptual judgement and motor response processes that are both activated in the frontal ROIs. Spatio-temporal heterogeneity in the frontal regions seems to be associated with diverse dynamic localizations of the two hidden processes in different subregions of frontal ROIs.

## Highlights

• We present a “spatio-temporal prototype”-based approach to fMRI data analysis.
• Our model is capable of disentangling overlapping mental processes evoked by stimuli.
• We found frontal ROIs were functionally less homogeneous than occipitotemporal ones.
• We detected spatio-temporal heterogeneity of mental processes in the frontal regions.

## Introduction

Since the first report of the Blood Oxygen Level-Dependent (BOLD) effect in humans, fMRI has been established as a powerful tool to non-invasively study the link between cognitive processes and the haemodynamic (BOLD) response that indirectly reflects evoked neuronal activity (Ogawa et al., 1990). Because of the limitation in sampling resolution and signal-to-noise ratio, statistical analysis of fMRI data plays an important role in revealing this relationship (Friston, 2005, Lindquist, 2008).

In particular, the primary aim of fMRI data analysis is the detection of activated brain areas in response to given stimulus types. This is intrinsically related to estimation of the underlying temporal dynamics, usually referred to as characterisation of the Haemodynamic Response Function (HRF). Detection of brain activation requires specification of a HRF shape throughout the brain. Due to low sampling resolution and poor signal-to-noise ratio, an accurate estimation of HRF shapes is only available from a group of voxels eliciting signal fluctuations correlated with the paradigm, usually referred to as region of interest (ROI). Thus, only spatio-temporal modelling of fMRI data can account for the relationship between a stimulus (or cognitive task) and the cortical response measured with fMRI (Derado et al., 2010, Gossl et al., 2001, Penny et al., 2005, Woolrich et al., 2004b).

A standard approach to spatio-temporal modelling of fMRI data spatially constrains (e.g. through Markov random field) mass univariate methods that model fMRI time series in individual voxels (Bai et al., 2009, Flandin and Penny, 2007, Friston et al., 2003, Kay et al., 2008, Penny et al., 2005, Penny et al., 2006, Svensen et al., 2000, Woolrich et al., 2004b). As an alternative to spatially constraining individual voxel-based models, spatial mixing of several localized ‘prototypical’ univariate models has been considered (Hartvig and Jensen, 2000, Kim et al., 2010, Penny and Friston, 2003, Vincent et al., 2010). In comparison to the former approach, the latter one is computationally more efficient (small number of free parameters) and yields more interpretable models (each prototype can correspond to an underlying source of neural activation triggered by the stimulus). In this contribution we propose a new method for spatio-temporal modelling of fMRI data that advances the latter approach in four crucial aspects:

1. Previously, the localized temporal prototypes have mostly been General Linear Models (GLMs) (Friston et al., 1995) (see e.g. Penny and Friston, 2003; Kim et al., 2010; Vincent et al., 2010), which could be relatively simple (the onset and shape of HRF are assumed to be known and remain the same across all prototypes/voxels). Instead, we use as prototypes Hidden Process Model (HPM) (Hutchinson et al., 2009), which enables us to infer the contribution of individual cognitive processes to the observed fMRI data. As in HPM, the onset times of HRF are allowed to vary. Crucially, we use parameterized forms of hidden processes, thus imposing biological constraints on the form of the HRF (which can differ for each cognitive process).
2. Recently, in cognitive science the investigation of inter-sessional variations of temporal patterns (in addition to variations across ROIs) has gained prominence (Duff et al., 2007, Mayhew et al., 2012). Unlike the previously mentioned methods, our approach can provide a complete, yet sparse representation of spatio-temporal patterns of neural activation within individual ROIs.
3. Whereas all previous approaches have been validated on data from block-design experiments, we devise a robust learning algorithm that enables our approach to be used in modelling data coming from relatively rapid event-related experimental designs.
4. As in Penny and Friston (2003), our model is a probabilistic model of the data and so crucial properties, such as the number and location of the underlying sources of neural activation (prototype number and positions in the voxel space), can be inferred in a principled manner. To determine the number of prototypes we have developed an MCMC algorithm to compute the model evidence.

In general, prototype models for spatio-temporal analysis of fMRI data are based on the assumption that the spatio-temporal behaviour of fMRI data could be characterised by a small set of temporal patterns that spread locally around sources (prototypes) in the voxel space. This assumption could be rationalised by the well known fact that the neural activation triggered by external stimuli usually has multiple latent sources which are spatially well localized. The prototypes of temporal patterns could be considered as cognitive signals originating from those sources and fMRI data are generated by the superposition of these signals. In this work, the temporal pattern and spatial spread of each prototype are modelled separately, but a parametric approach is adopted in both cases. However, the temporal and spatial aspects of our model are not independent, since they are integrated into a unified spatio-temporal model through a spatially regularized mixture. Within this framework, the problem of activation detection is simply rendered as an estimation problem if the number of latent sources is known. Otherwise, model selection for mixture models provides a principled way to determine this number.

One of the most widely used methods for fMRI data analysis is the so-called Statistical Parametric Maps (SPM), introduced by Friston et al. (1995). In SPM, not only the spatial and temporal aspects of fMRI model are treated separately, but also the analysis is split into two steps. In the first step, General Linear Models (GLMs) are fitted to fMRI time series. Regressors of the GLM (columns of its design matrix) represent the models' assumptions about the haemodynamic response evoked by stimulation.^1^ Therefore, only GLM regression coefficients are estimated from the data. In the second step, the estimated coefficients are tested against a particular hypothesis in order to detect the activation. The essential difference between SPM and our approach is two-fold: 1) from the data we infer not only the response magnitudes but also response shapes, together with response onsets; and 2) the task of activation detection is done naturally in one step and in a model based manner.

A variety of approaches have been suggested in the literature to model and estimate HRFs (Bai et al., 2009, Friston et al., 2003, Kay et al., 2008, Svensen et al., 2000, Woolrich et al., 2004b). They can be broadly grouped into parametric, non-parametric, and semi-parametric approaches. In a parametric approach, HRF is represented by an analytical function with a small set of free parameters to be learned from the data. In a non-parametric approach, the entire function or its values at discretised times are to be estimated (FIR model). As this estimation problem is obviously ill-posed, some smoothness constraints need to be imposed (Tikhonov regularization (Casanova et al., 2008, Kay et al., 2008), Gaussian process prior (Marrelec et al., 2003, Zhang et al., 2008)). In a semi-parametric approach, the HRF is modelled using a small set of basis functions (Woolrich et al., 2004a). In our work, we adopt a parametric approach to HRF modelling. To our knowledge, this approach has not yet been applied to fMRI data from rapid event-related experiments. Also, the temporal model adopted in those studies is relatively simple as 1) a single process is used to describe the haemodynamic response to stimuli; and 2) the process onsets are assumed to be known. However, a stimulus can trigger a number of different cognitive processes, that is, visual analysis process, perceptual judgement process, and motor-response process. These processes need to be represented individually in the temporal model. The temporal model adopted in our work is very similar to that adopted in previous studies (Hutchinson et al., 2009). However, the non-parametric approach is adopted in that work. Further, we used a rapid event-related design (Mayhew et al., 2012) in contrast with previous work using long trials that may allow easier separation of cognitive processes (Hutchinson et al., 2009).

Spatial priors are often used to extend a mass-univariate model such as GLM to a fully Bayesian spatio-temporal model for fMRI data (Flandin and Penny, 2007, Penny et al., 2005, Penny et al., 2006). As mentioned above, a common strategy is to impose a Markov random field (MRF) prior on GLM regression coefficients (Gossl et al., 2001, Penny et al., 2005), or on the estimates of HRFs (Hutchinson et al., 2009). In cases where model residuals are treated as auto-regressive (AR) time series, MRF priors are also imposed on AR parameters (Woolrich et al., 2004b). An alternative to MRF is the so-called spatial mixture model (SMM) approach. Initially, SMM was applied to activation detection by fitting a mixture of three-dimensional Gaussian functions to those statistical parametric maps from GLM analysis (Kim et al., 2010). Recently, the SMM approach has been further developed towards a spatio-temporal model of fMRI data, that is, a spatially regularized mixture model of several GLM components. Examples are: a mixture of several GLMs with different, but fixed design matrices (Penny and Friston, 2003) and a Gaussian mixture model for the prior of GLM regression coefficients (Hartvig and Jensen, 2000, Vincent et al., 2010). Compared to these previous studies, our approach allows not only different response magnitudes but also varying HRF shapes across the mixture components. Both magnitudes and shapes are to be estimated from the data.

The paper is organised as follows. After a brief introduction to spatio-temporal modelling of fMRI data (Introduction section), we formulate our model and describe a numerical algorithm to learn model parameters in Methods section. In Results section, the validation of our approach is presented using both synthetic and real data. The paper is concluded with discussion in Discussion section.

## Methods

### Spatio-temporal modelling

Let a fMRI data set of *V* voxels and *T* volume (time steps) be denoted by a matrix $Y\in R^{V\times T}$, a fMRI time series at voxel *v* by a vector $y\left(v\right)\in R^{T}$, a fMRI measurement at voxel *v* and time *t* by a scalar *y*(*v*,*t*).

Assume that *K* characteristically different and spatially localized temporal patterns could be observed in **Y**. To formulate a spatio-temporal model for **Y**, we first define the likelihood of *y*(*v*,*t*) as follows

(1) $$p\left(y\left(v,t\right)\right)=\sum_{k=0}^{K}\;p\left(k|v\right)\cdot p\left(y\left(v,t\right)|k\right),$$

where index *k* here represents a temporal model that could explain the *k*-th temporal pattern observed in **Y**. The probability *p*(*k*|*v*) is the prior probability for the *k*-th model being chosen to generate fMRI time series *y*(*v*) at voxel *v* and *p*(*y*(*v*,*t*)|*k*) is the probability for *y*(*v*,*t*) being predicted by model *k*. Non-zero indices *k* represent models that account for prototypical patterns originating from some spatially localized sources of neural activation; *k* = 0 indexes of a null model accounting for temporal patterns that are not related to any neural activation.

The above definition could be rationalised by the fact that a small number of prototypical temporal patterns is often observed in a particular ROI. At some voxels, one of those patterns can be clearly recognised while the time series in other voxels resemble several patterns to different degrees, which vary smoothly across the regions of interest.

The definition of *p*(*y*(*v*,*t*)) in Eq. (1) represents a space–time separation approach to spatio-temporal modelling. It is clear that given a voxel indexed by *v*, the probability *p*(*k*|*v*) is independent of time index *t*. The density *p*(*y*(*v*,*t*)|*k*) is actually the likelihood function of model *k* evaluated at *y*(*v*,*t*). Note that this likelihood function itself, *p*(*y*|*k*), is independent of voxel index *v*. Let *Θ*^STM^ denote a parameter set of the above model. Obviously, this set comprises of a set of spatial parameters and a set of temporal parameters, denoted by $\Theta^{S}$ and $\Theta^{T}$, which specify the probabilities *p*(*y*(*v*,*t*)|*k*) and *p*(*k*|*v*), respectively. The definition of $p\left(y\left(v,t\right)|k,\Theta^{T}\right)$ and $p\left(k|v,\Theta^{S}\right)$ is given in the Introduction and Spatial modelling, Introduction and Spatial modelling sections, respectively.

### Temporal modelling

**Fig. 1:**
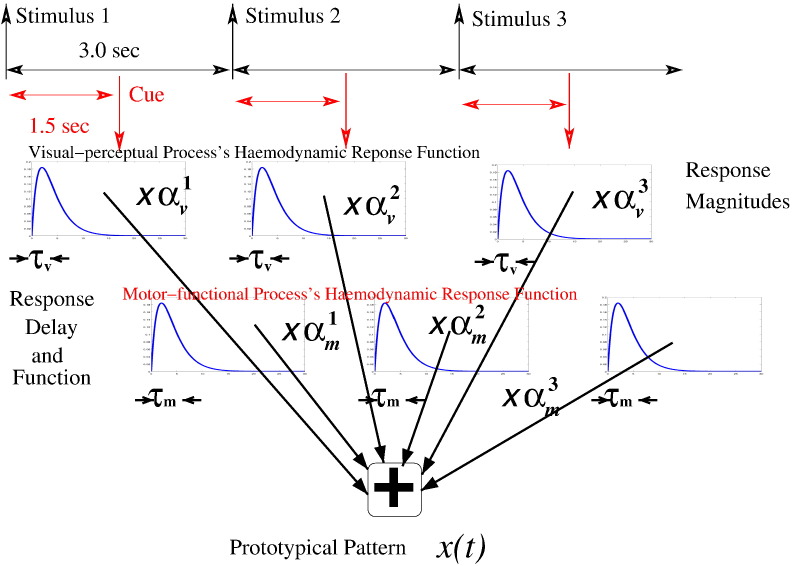
Illustration of parametric temporal model.

Our temporal model of fMRI time series is schematically illustrated in Fig. 1. In this model, the haemodynamic response of every single stimulus breaks down into its constituents, that is, the haemodynamic response of individual cognitive processes evoked by that stimulus. This represents a new approach to haemodynamic response modelling and is firstly proposed in Hutchinson et al. (2009).

As the temporal models are independent of voxel index *v*, they are considered as parametric model for *y*(*t*). Further, it is assumed that except for the model with *k* = 0, all temporal models share a canonical form. This canonical model is given as follows:

• A fMRI time series *y*(*t*) is composed of a signal component *x*(*t*) and a noise component *∈*(*t*), i.e.
  $$y\left(t\right)=x\left(t\right)+\epsilon \left(t\right);$$
• The noise component *∈*(*t*) is modelled by white Gaussian noise with noise variance *σ*^2^, i.e.
  $$\epsilon \left(t\right)∼N\left(0,\sigma^{2}\right).$$
  We note that the assumption of i.i.d. noise can cause enhanced false-positive rate in activation detection. However, as pointed out in Heller et al. (2006) and Penny and Friston (2003), clustering-based methods (such as ours) are typically much less prone to false positives caused by the neglect of autocorrelation in fMRI noise;
• The signal component *x*(*t*) is given by
  $$x\left(t\right)=\sum_{s=1}^{S}\sum_{p=1}^{P}h_{p,s}\left(t\right),$$
  where S is the total number of stimuli in a time window, *P* is the number of cognitive processes evoked by a stimulus, and **h**_**p**,*s*_(*t*) represents the haemodynamic response of the **P**-th process evoked by the *s*-th stimulus;
• The haemodynamic response **h**_**p**,*s*_(*t*) is given by
  $$h_{p,s}\left(t\right)=a_{p,s}\cdot \delta \left(t-\left(t_{p,s}+\tau_{p,s}\right)\right)⊗g_{p,s}\left(t\right),$$
  where *a*_**p**,*s*_ is response magnitude, *t*_**p**,*s*_ is response onset, *τ*_**p**,*s*_ is response delay, and *g*_**p**,*s*_(*t*) represents response shape function. Moreover, *δ*() denotes delta function and ⊗ denotes convolution operator. As adopted by Liao et al. (2002), we also use a time-shift model to account for the delay of the fMRI responses. Note that Liao et al. (2002) did make a first-order Taylor approximation to the time-shift model to transform a non-linear estimation problem into a linear one. We don't make such approximation;
• The response shape function *g*_**p**,*s*_(*t*) is defined as a Gamma function *g*(*t*) with its shape parameter *κ*_**p**,*s*_ and scale parameter *θ*_**p**,*s*_, i.e.
  $$g_{p,s}\left(t\right)=g\left(t|\kappa_{p,s},\theta_{p,s}\right)=\frac{t^{\kappa_{p,s}-1}\exp\left(-\frac{t}{\theta_{p,s}}\right)}{{\left(\theta_{p,s}\right)}^{\kappa_{p,s}}\Gamma \left(\kappa_{p,s}\right)}.$$
  The gamma function was firstly proposed as a canonical HRF in Svensen et al. (2000).

We denote all haemodynamic response parameters by $\Theta_{h}^{T}$, that is,

$$\Theta_{h}^{T}={\left\{a_{p,s},\tau_{p,s},\theta_{p,s},\kappa_{p,s}\right\}}_{\begin{array}{cc} p=1,\ldots ,P & s=1,\ldots ,S \end{array}}.$$

Note that response onset *t*_**p**,*s*_ is a known parameter and $\Theta_{h}^{T}$ is a 4·*S*·*P*-dimensional vector of free parameters. As we have *K* temporal models of this canonical form, the *k*-th model is specified by its parameter set $\Theta_{h,k}^{T}$ and noise parameter *σ*_*k*_^2^. Its signal component is given by

$$x_{k}\left(t\right)=x\left(t;\Theta_{h,k}^{T}\right),$$

and the corresponding likelihood is

$$p\left(y\left(v,t\right)|k;\Theta_{k}^{T}\right)=N\left(y\left(v,t\right);x_{k}\left(t\right),\sigma_{k}^{2}\right),$$

with $\Theta_{k}^{T}=\left(\Theta_{h,k}^{T},\sigma_{k}^{2}\right)$ for *k* ≠ 0.

For the null model (*k* = 0), we have

$$\begin{array}{ccc} x\left(t\right)=b+\epsilon \left(t\right) & \mathrm{with} & \epsilon \left(t\right)∼N\left(0,\sigma_{0}^{2}\right), \end{array}$$

which accounts for a possible level shift of fMRI signal. Moreover, the shift is assumed to be constant over time. The corresponding likelihood is given by

$$p\left(y\left(v,t\right)|k=0;\Theta_{0}^{T}\right)=N\left(y\left(v,t\right);b,\sigma_{0}^{2}\right),$$

with $\Theta_{0}^{T}=\left(b,\sigma_{0}^{2}\right)$.

**Fig. 2:**
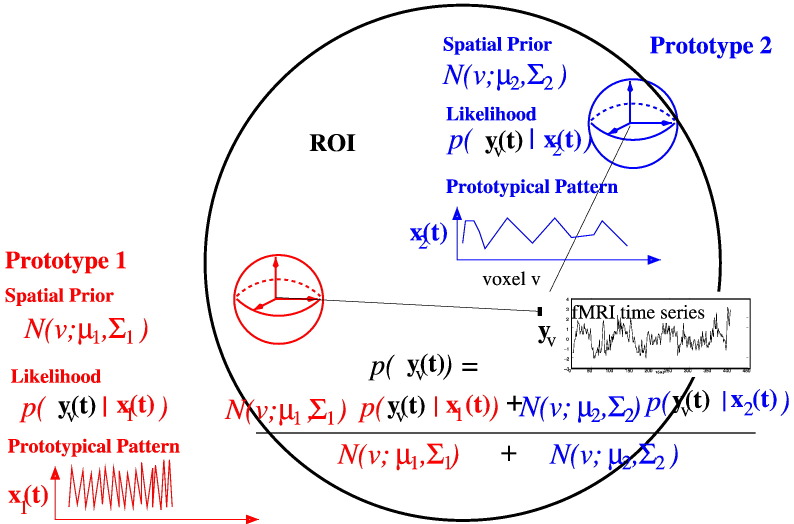
Illustration of spatial mixture model.

In summary, the set of temporal parameters $\Theta^{T}=\left\{\Theta_{0}^{T},\Theta_{1}^{T},\ldots ,\Theta_{K}^{T}\right\}$. includes totally *K*·(4·*S*·*P* + 1) + 2 free parameters: 4·*K*·*P*·*S* haemodynamic response parameters, 1 level shift parameter, and *K* + 1 noise parameters (Fig. 2).

### Spatial modelling

As pointed out in the previous subsection, the prior probability *p*(*k*|*v*) varies across the regions of interest. Clearly, it is an ill-posed problem to estimate *p*(*k*|*v*) for every *v*. More importantly, it is known that evoked neural responses are spatially contiguous. Therefore, it is natural to impose smoothness constraints on the spatial variation of *p*(*k*|*v*).

Recall that $\Theta^{S}$ denotes the set of spatial parameters that specify the spatial prior *p*(*k*|*v*). Note that Given voxel *v*, this prior probability is defined by the likelihood ratio

$$p\left(k|v;\Theta^{S}\right)=\frac{p\left(v|k;\Theta_{k}^{S}\right)}{\sum_{k=0}^{K}p\left(v|k;\Theta_{k}^{S}\right)},$$

where $p\left(v|k;\Theta_{k}^{S}\right)$ is the likelihood of model *k* of “influence” having voxel *v* in its “region of influence”. In contrast, $p\left(k|v;\Theta^{S}\right)$ is the probability of voxel *k* “belonging” to model *k*, (**y**(*t*) = *x*_*k*_(*t*)). Note that we have $\Theta^{S}=\left\{\Theta_{0}^{S},\Theta_{1}^{S},\ldots ,\Theta_{K}^{S}\right\}$. This definition allows the smoothness constraints to be placed on *p*(*v*|*k*) while ensuring that ∑_*k* = 0_^*K*^*p*(*k*|*v*) = 1.

Assume that the haemodynamic response of a certain neural activation propagates from an epi-centre across the whole ROIs with certain covariance structure. Mathematically, this could be modelled by a three-dimensional Gaussian distribution. Hence, the likelihood is given by

(2) $$p\left(v|k\right)=N\left(r_{v}|\mu_{k},\Sigma_{k}\right),$$

where **r***_v_* denotes the location of voxel *v*, *μ_k_* is the mean vector of the Gaussian distribution, and Σ*_k_* is its covariance matrix. Note that we have $\Theta_{k}^{S}=\left(\mu_{k},\Sigma_{k}\right)x$ for *k* ≠ 0.

For the null model (*k* = 0), we have $p\left(v|k=0\right)=\frac{1}{V},$ where *V* is a free normalization parameter (i.e. $\Theta_{0}^{S}=V$). This definition is rationalised by the assumption that the level shift of BOLD signals stays constant across individual ROIs. Note that *V* ought to take a value larger than *V* (the number of voxels in a ROI). Otherwise, the null model could often dominate over the other models. This is because the spatial extent of ROIs is bounded and the probability mass of *p*(*v*|*k*) over some ROIs could be significantly smaller than 1.

In summary, the set of spatial parameters $\Theta^{S}$ includes totally 9·*K* + 1 free parameters: 3·*K* mean parameters, 6·*K* covariance parameters, and 1 normalization parameter.

### The posterior

In this work, a Bayesian approach is adopted to estimate all model parameters, i.e. *Θ*^STM^ that are used to specify our spatio-temporal model of fMRI data by maximizing the posterior distribution

$$p\left(\Theta^{\mathrm{STM}}|Y\right)=p\left(Y|\Theta^{\mathrm{STM}}\right)\cdot p\left(\Theta^{\mathrm{STM}}\right)$$

where likelihood *p*(**Y**|*Θ*^STM^) and prior *p*(*Θ*^STM^) are specified in what follows.

Given our model, all fMRI measurements are conditionally independent in both spatial and temporal domains. Therefore, we have

$$\begin{array}{l} \;p\left(Y|\Theta^{\mathrm{STM}}\right)\\ \begin{array}{cc} = & \prod_{v}\prod_{t}p\left(y\left(t,v\right)|\Theta^{T},\Theta^{S}\right) \end{array}\\ \begin{array}{cc} = & \prod_{v}\prod_{t}\sum_{k=0}^{K}p\left(k|v;\Theta^{S}\right)\cdot p\left(y\left(t,v\right)|k;\Theta_{k}^{T}\right) \end{array}\\ \begin{array}{cc} = & \prod_{v}\prod_{t}\frac{\sum_{k=0}^{K}p\left(v|k;\Theta_{k}^{S}\right)\cdot p\left(y\left(t,v\right)|k;\Theta_{k}^{T}\right)}{\sum_{k=0}^{K}p\left(v|k;\Theta_{k}^{S}\right)} \end{array}\\ \begin{array}{cc} = & \prod_{v}\prod_{t}\left\{\begin{array}{l} \frac{\frac{1}{V}\cdot N\left(y\left(v,t\right);b,\sigma_{0}^{2}\right)}{\sum_{k=1}^{K}N\left(r_{v}|\mu_{k},\Sigma_{k}\right)+\frac{1}{V}}\\ +\frac{\sum_{k=1}^{K}N\left(r_{v}|\mu_{k},\Sigma_{k}\right)\cdot N\left(y\left(v,t\right);x\left(t;\Theta_{h,k}^{T}\right),\sigma_{k}^{2}\right)}{\sum_{k=1}^{K}N\left(r_{v}|\mu_{k},\Sigma_{k}\right)+\frac{1}{V}} \end{array}\right\} \end{array}. \end{array}$$

Recall that $\Theta_{h,k}^{T}$ represents a set of haemodynamic response parameters that is used to specify the *k* temporal model.

Finally, the prior *p*(*Θ*) is factorized as follows:

$$p\left(b\right)\cdot \left(\prod_{k=1}^{K}p\left(\Theta_{h,k}^{T}\right)\right)\cdot \left(\prod_{k=1}^{K}p\left(\sigma_{k}^{2}\right)\right)\cdot p\left(V\right)\cdot \left(\prod_{k=1}^{K}p\left(\mu_{k}\right)p\left(\Sigma_{k}\right)\right).$$

We further assume the same prior on $\Theta_{h,k}^{T}$ for all *k* ≠ 0, i.e. $p\left(\Theta_{h,k}^{T}\right)=p\left(\Theta_{h}^{T}\right)$ which can be factorized as follows:

$$\prod_{s=1}^{S}\prod_{p=1}^{P}p\left(a_{p,s}\right)\cdot p\left(\tau_{p,s}\right)\cdot p\left(\theta_{p,s},\kappa_{p,s}\right).$$

For some parameters such as **b**, *V*, *a*_**p**,*s*_, and *μ_k_*, no prior information is available because of large variability across a pool of fMRI data sets. Hence, their prior is set to a uniform distribution. For the rest of the parameters, we assume that the same prior should apply to all parameters of the same type, for instance, all noise parameters across prototypes. Therefore, the corresponding indices (e.g. *k* for the noise parameters) are dropped in the remaining of this subsection.

For the variance parameter *σ*^2^, its likelihood profile is normally flat for large *σ*^2^. To make the estimation of this parameter robust, its prior is set to $p\left(\sigma^{2}\right)\propto \frac{1}{{\left(\sigma^{2}\right)}^{2}}$. Similarly, the prior of a covariance matrix (Σ) is set to the so-called Jeffery prior, i.e. $p\left(\Sigma \right)\propto \frac{1}{{\left|\Sigma \right|}^{2}}$ where |*Σ*| is the determinant of Σ.

For the response delay parameter *τ*, it is found in the previous EEG-informed fMRI study (Mayhew et al., 2012) that *τ* varies roughly between 0.1 s and 0.3 s. Hence, a Gaussian distribution is used to represent this prior knowledge, with its mean equal to 0.2 s and its variance equal to 0.01. For good understanding of this time scale, we note that the time interval between two subsequent measurements is 1.5 s.

For the response shape parameter *κ* and *θ*, we make use of its relation to so-called time-to-peak parameter *T* and full-width-at-half-maximum parameter *W* of a Gamma function as follows T = (*κ* − 1)*θ* and $W=\sqrt{2\ln2}\cdot \sqrt{\kappa }\theta$, respectively. It is reasonable to assume that the latency and duration of a haemodynamic response have an upper bound: *T_max_* = 4 s and *W_max_* = 8 s (Friston et al., 1994, Friston et al., 1995). Thus, a logarithmic barrier function is used to represent this prior knowledge about the shape and scale parameter, that is,

$$p\left(\kappa ,\theta \right)\propto \exp^{-\log\left(T_{\max}-T\right)-\log\left(W_{\max}-W\right)}.$$

### Generative model

In general, clustering fMRI time series in different voxels doesn't provide a generative model. As shown in Fig. 1 and Fig. 2, however, our clustering-like spatio-temporal model is a generative model. Therefore, the synthetic data can be generated by simulating the model with the parameters that are specified as above. The simulation is split into 3 steps:

1. Generate the corresponding prototypical fMRI time series *x_k_*(*t*) for each prototype *k*;
2. Compute the corresponding weight distribution *p*(*k*|*v*) for each voxel *v*;
3. Generate synthetic fMRI time series at voxel *v* as $y\left(t,v\right)=x_{k_{t}}\left(t\right)$ where *k_t_* are i.i.d. random samples drawn from *p*(*k*|*v*).

### Gradient-based learning

As seen in the previous two subsections, we have two subsets of model parameters to be learned from the data, those in temporal and spatial models. They are ${\left\{\Theta_{k}^{T}\right\}}_{k=1}^{K}$ and ${\left\{\Theta_{k}^{S}\right\}}_{k=1}^{K}$ respectively. In this work, these 2 subsets of parameters are optimized iteratively. For each subset, a scaled conjugate-gradient optimization algorithm is employed.

It is worth to interpret the gradients of model parameters, although their full expression is not given. To that end, we first define the posterior probability of the model index *k* given the data *y*(*t*,*v*) as follows

$$p\left(k|y\left(t,v\right);{\hat{\Theta }}^{\mathrm{STM}}\right)=\frac{p\left(k|v;{\hat{\Theta }}^{S}\right)\cdot p\left(y\left(t,v\right)|k;{\hat{\Theta }}_{k}^{T}\right)}{\sum_{\tilde{k}=0}^{K}p\left(\tilde{k}|v;{\hat{\Theta }}^{S}\right)\cdot p\left(y\left(t,v\right)|\tilde{k};{\hat{\Theta }}_{\tilde{k}}^{T}\right)},$$

where we use the current parameter set

$${\hat{\Theta }}^{\mathrm{STM}}=\left\{{\hat{\Theta }}^{T},{\hat{\Theta }}^{S}\right\}={\left\{{\hat{\Theta }}_{k}^{T},{\hat{\Theta }}_{k}^{S}\right\}}_{k=1}^{K}.$$

This probability is also seen as the responsibility of the model indexed by *k* for explaining the data *y*(*t*,*v*).

For the parameter vector $\Theta_{k}^{T}$ of the *k*-th temporal model, we have

$$\nabla_{\Theta_{k}^{T}}\left\{-\mathrm{logp}\left(\Theta^{\mathrm{STM}}|Y\right)\right\}=\sum_{v=1}^{V}\sum_{s=1}^{S}\left\{\begin{array}{l} p\left(k|y\left(t,v\right);{\hat{\Theta }}^{\mathrm{STM}}\right)\\ \cdot \nabla_{\Theta_{k}^{T}}\left\{-\mathrm{logp}\left(y\left(t,v\right)|k,\Theta_{k}^{T}\right)\right\}{|}_{\Theta_{k}^{T}={\hat{\Theta }}_{k}^{T}} \end{array}\right\}.$$

This shows that the gradient of the negative log posterior probability is a weighted sum of the gradients of the negative log prediction probability for every single fMRI measurement *y*(*t*,*v*) while the weights are the corresponding responsibilities for the *k* model.

For the spatial parameter vector $\Theta_{k}^{S}$, we have

$$\begin{array}{l} \nabla_{\Theta_{k}^{S}}\left\{-\mathrm{logp}\left(\Theta^{\mathrm{STM}}|Y\right)\right\}=\sum_{v=1}^{V}\;\sum_{s=1}^{S}\;\left\{\left(p\left(k|y\left(t,v\right);{\hat{\Theta }}^{\mathrm{STM}}\right)-p\left(k|v;{\hat{\Theta }}^{S}\right)\right)\right)\\ \;\left(\cdot \nabla_{\Theta_{k}^{S}}\left\{-\mathrm{logp}\left(v|k,\Theta_{k}^{S}\right)\right\}{|}_{\Theta_{k}^{S}={\hat{\Theta }}_{k}^{S}}\right\}. \end{array}$$

This shows that the gradient of the negative log posterior probability is a weighted sum of the gradients of the negative log spatial prior for every single fMRI measurement *y*(*t*,*v*). The weights here are the difference between the posterior and the prior probability for model *k* being chosen to explain *y*(*t*,*v*), which reflects the fact that updating of spatial priors is guided by how well the prior matches the actual distribution of fMRI time series.

### Model initialisation

For any gradient-based optimization algorithms, only local optimum could be reached. The posterior distribution of a mixture-of-experts model could be highly multi-modal. Therefore, a good initialisation is crucial. In this work, we adopt a data-driven approach to initialise our model's spatial parameters and a greedy approach to initialise its temporal parameters.

First, the prototypes are (roughly) identified by clustering fMRI time series with a *K*-means algorithm. In general, *K*-means clustering can be rather sensitive to initialisation. Other more robust clustering techniques could be used, e.g. Neural Gas (Fritzke, 1995, Martinetz et al., 1993).^2^ However, we use small codebook sizes (up to 4) and in such cases *K*-means with codebook vectors initialised in randomly picked training points is more robust to initialisation than in the case of larger codebooks. We have adopted a multiple random initialisation approach — clustering with different random initialisations is repeated 50 times and the clustering solution with minimum distortion measure is accepted.

After all voxels are grouped into *K* clusters, a good guess of the spatial parameters can be obtained by computing the mean vector and covariance matrix of each prototype from the coordinates of voxels in the corresponding cluster. Similarly, the temporal parameters of each prototype can be initialised by fitting the corresponding temporal model to the fMRI time series from those voxels in the corresponding cluster.

Both the clustering step and the greedy step could be repeated several times to obtain better initialisation. At each iteration, we generate a time series for each of the *K* temporal models and use these time series as the initialisation for *K*-means clustering.

The above algorithm is based on the assumption that the number of prototypes *K* is known. An extension of this algorithm is proposed to obtain a good, fast initialisation of *K* + 1 and *K* − 1 prototypes from the available *K* prototypes by using the so-called birth and merge operations described as follows:

*Birth operation*. A new prototype is needed if there is a group of voxels that are not well accounted for by the current model. To identify those voxels, a cross-validation approach is adopted. In order to do this, a subset of voxels that are to be pruned out is chosen randomly. Prediction probabilities of the fMRI time series **y**(***v***) at those voxels are computed as
  $$\mathrm{Pred}\left(y\left(v\right)\right)=\prod_{t}p\left(y\left(t,v\right)|{\hat{\Theta }}^{T},{\hat{\Theta }}^{S}\right).$$
  Recall that ${\hat{\Theta }}^{T}$ and ${\hat{\Theta }}^{S}$ denote the current temporal and spatial parameters, respectively. If there exists a group of voxels with lower Pred (**y**(*v*)) and they are also spatially contiguous, we add a new prototype, representing the spatio-temporal pattern across those voxels, to the current model. The temporal and spatial parameters of this prototype are initialised in the same way as those of other prototypes are initialised after *K*-means clustering of fMRI time series;
*Merge operation*. To merge a pair of two prototypes, we compute so-called responsibility vector for each prototype as
  $$\gamma_{k}={\left[\sum_{t=1}^{T}\;p\left(k|y\left(t,1\right);{\hat{\Theta }}^{\mathrm{STM}}\right),\ldots ,\sum_{t=1}^{T}\;p\left(k|y\left(t,V\right);{\hat{\Theta }}^{\mathrm{STM}}\right)\right]}^{⊤}$$
  and the (normalized) similarity measure *d_ij_* between two prototypes *i* and *j* is given by
  $$d_{\mathrm{ij}}=\frac{\gamma_{i}\gamma_{j}^{⊤}}{\gamma_{i}\gamma_{i}^{⊤}\cdot \gamma_{j}\gamma_{j}^{⊤}}.$$
  The larger *d*_*ij*_ ∈ [− 1, 1] is, the more overlapping these two clusters are. The mean *μ*^new^ and covariance matrix Σ^new^ of the resulting merged prototype are obtained as follows: *μ*^new^ = *π*_*i*_*μ*_*i*_ + *π*_*j*_*μ*_*j*_ and
  $$\Sigma^{\mathrm{new}}+\mu^{\mathrm{new}}{\left(\mu^{\mathrm{new}}\right)}^{⊤}=\pi_{i}\left(\Sigma_{i}+\mu_{i}\mu_{i}^{⊤}\right)+\pi_{j}\left(\Sigma_{j}+\mu_{j}\mu_{j}^{⊤}\right)$$
  with the weights $\pi_{i}=\frac{\gamma^{i}}{\gamma^{i}+\gamma_{j}}$ and $\pi_{j}=\frac{\gamma^{j}}{\gamma^{i}+\gamma_{j}}$, where *γ*^*i*^ and *γ*^*j*^ are computed as *γ*^*i*^ = ∑_*k*_ *γ*_*i*_(*k*) and *γ*^*j*^ = ∑ _*k*_ *γ*_*j*_(*k*), respectively.

Note that the birth and merge operations described above are related to the SMEM algorithm (Ueda et al., 2000).

### Model selection

In practice, the number of components *K* in a mixture model is unknown. In our case, the number of prototypes required to explain fMRI data needs to be learned from the data. In a fully Bayesian setting, so-called Reversible Jump Markov Chain Monte Carlo (RJMCMC) (Richardson and Green, 1998) is a principled computational method to obtain a MAP estimate of *K*. An alternative approach is to consider the determination of the number of prototypes as a model selection problem. The criterion for model selection is so-called model evidence (Berkhof et al., 2003). In this work, a relative estimate of model evidence is computed for a number of *K*s with *K* > 1 relative to *K* = 1. To jointly compute those estimates, we use so-called Wang–Landau algorithm (Atchad and Liu, 2010) that is based on controlled Markov chains. For the above purpose, this algorithm has better convergence properties than other cross-dimensional MCMC algorithms.

For model M with model parameter set *Θ*, model evidence is defined as

$$p\left(Y|M\right)=\int p\left(Y|\Theta ,M\right)p\left(\Theta |M\right)d\Theta$$

where $p\left(\Theta |M\right)$ is the prior on *Θ* and $p\left(Y|M,\Theta \right)$ is the likelihood of data **Y** under the model M. Considering two competing models $M_{1}$ and $M_{2}$, the so-called Bayes factor,

$$BF_{12}=\frac{p\left(Y|M_{1}\right)}{p\left(Y|M_{2}\right)},$$

is computed and if this number is larger than 1, then $M_{1}$ has a higher posterior probability, and vice versa.

To compute the Bayes factor *BF*_12_, one can sample from both posteriors $p\left(\Theta_{1}|Y,M_{1}\right)$ and $p\left(\Theta_{2}|Y,M_{2}\right)$. Those samples can be used to compute $p\left(Y|M_{1}\right)$ and $p\left(Y|M_{2}\right)$. However, the estimates could be very inaccurate for the determination of *BF*_12_. A more efficient way to compute *BF*_12_ is the so-called acceptance-ratio method (Bennett, 1976) in which one does sample from the joint posterior $p\left(\Theta_{M},M|Y\right)=p\left(M\right)\cdot p\left(\Theta_{M}|Y,M\right),$ where $p\left(M\right)$ is the prior of model M. This can be done by any MCMC algorithm which allows moves between $M_{1}$ and $M_{2}$. By the detailed balance requirement of a MCMC algorithm, we have

$$p\left(M_{1}\right)\cdot p\left(\Theta_{1}|Y,M_{1}\right)\cdot T\left(\Theta_{1}\to \Theta_{2}\right)\;=\;p\left(M_{2}\right)p\left(\Theta_{2}|Y,M_{2}\right)\cdot T\left(\Theta_{2}\to \Theta_{1}\right),$$

where **T**(*Θ*_1_ → *Θ*_2_) is the transition kernel that allows a move from $M_{1}$ to $M_{2}$ and vice versa. By integrating both sides of detailed balance equation with respect to *Θ*_1_ and *Θ*_2_, it follows

$$\frac{p\left(Y|M_{2}\right)}{p\left(Y|M_{1}\right)}=\frac{p\left(M_{2}\right)}{p\left(M_{1}\right)}\cdot \frac{E_{\Theta_{1}}\left(T\left(\Theta_{1}\to \Theta_{2}\right)\right)}{E_{\Theta_{2}}\left(T\left(\Theta_{2}\to \Theta_{1}\right)\right)}.$$

It can be seen that

$$\frac{p\left(Y|M_{2}\right)}{p\left(Y|M_{1}\right)}=\frac{p\left(M_{2}\right)}{p\left(M_{1}\right)}\to \frac{E_{\Theta_{1}}\left(T\left(\Theta_{1}\to \Theta_{2}\right)\right)}{E_{\Theta 2}\left(T\left(\Theta_{2}\to \Theta_{1}\right)\right)}=1.$$

The above derivation shows that an estimate of relative model evidence is obtained if the prior on M can be tuned so that the resulting marginal posterior of model index should be uniform. In the Wang–Landau algorithm, the prior distribution of model index is modified at every MCMC step by an additive change which is proportional to the difference between a flat histogram and the empirical histogram computed from a counter of the model indices that have been sampled from the posterior. Once the empirical histogram has become sufficiently flat, the counter is set to null and the proportional constant is reduced by a certain factor. These two steps shall be repeated until the estimate of *BF*_12_ has been stabilised.

In this work, the transition kernel **T**(*Θ*_1_ → *Θ*_2_) that allows a move from $M_{1}$ to $M_{2}$ is implemented by a RJMCMC algorithm with

$$T(\Theta_{2}\to \Theta_{1}=J\left(\Theta_{2}\to \Theta_{1}\right)\cdot \min\left\{1,\frac{p\left(M_{1}\right)}{p\left(M_{2}\right)}\cdot \frac{p\left(\Theta_{1}|Y,M_{1}\right)}{p\left(\Theta_{2}|Y,M_{2}\right)}\cdot \frac{J\left(\Theta_{2}\to \Theta_{1}\right)}{J\left(\Theta_{1}\to \Theta_{2}\right)}\right\},$$

where *J*(⋅→⋅) denotes a proposal density. To propose *Θ*_2_ by given *Θ*_1_, we have *Θ*_2_ = **f**(*Θ*_1_,**u**) where f denotes a deterministic function and u is a random vector drawn from some density, say *q*(u), which implies

$$\frac{J\left(\Theta_{2}\to \Theta_{1}\right)}{J\left(\Theta_{1}\to \Theta_{2}\right)}=\frac{\left|\nabla_{\Theta_{1},u}f\left(\Theta_{1},u\right)\right|}{q\left(u\right)}.$$

The RJMCMC algorithm in this work comprises two major ingredients, namely a birth proposal and a death proposal. To delete a prototype, one of the existing prototypes is randomly chosen. To propose a new prototype, the responsibilities of the null prototype are computed for every voxel *v* as follows

$$\pi_{v}^{0}=\frac{\sum_{t}\;p\left(0|Y_{v}^{t}\right)}{\sum_{k}\;\sum_{t}\;p\left(k|Y_{v}^{t}\right)}.$$

Also, compute *μ*^⁎^ and **Σ**^⁎^ as the weighted mean and covariance matrix of all voxels in this ROI and compute *Θ*_∗_^*t*^ by fitting our canonical temporal model into the fMRI time series from voxel *v*^∗^ = argmax_*v*_*π*_*v*_^0^. Following this, we draw a random sample *Θ* by

$$\Theta ∼N\left(\cdot |\Theta^{*}=\left(\Theta_{*}^{t},\mu^{*},\Sigma^{*}\right),\tilde{\Sigma }\right)$$

where $\tilde{\Sigma }$ is a predefined diagonal matrix that can be tuned to maximize acceptance ratio.

A more complete RJMCMC algorithm should include both splitting and merging proposals. In some cases such as ours, this could make the computation of proposal densities very complicated. In contrast, we have here $|\nabla_{\Theta_{1},u}f\left(\Theta_{1},u\right)|=1$ and $q\left(u\right)∼N\left(\cdot |0,\tilde{\Sigma }\right)$ because only birth and death moves are considered.

Between two RJMCMC steps, we also sample from $p\left(\Theta_{1}|Y,M_{1}\right)$ or $p\left(\Theta_{2}|Y,M_{2}\right)$, up to the current *K*-value, using a Hybrid Monte Carlo algorithm (Duane et al., 1987) which makes use of the gradients we have derived for our MAP algorithm.

## Results

In this section, we first present some results in a controlled experimental setting using synthetic data that validate the algorithm developed for estimating parameters of our spatio-temporal model. As our algorithm is a clustering-like method, it is worth noting that this approach is similar to so-called external measures in standard cluster validation (Halkidi et al., 2001). Following this, we apply our algorithm to real fMRI data obtained in a experiment designed to investigate which brain areas are involved in a shape discrimination task (i.e. discriminating radial from concentric patterns) (Mayhew et al., 2012). This task is known to engage occipitotemporal areas involved in the analysis of the visual stimuli and frontal regions engaged in perceptual judgements. Our model is assessed by its power to discriminate fMRI data from these two brain circuits.

### Description of fMRI data

All data sets we used in this study are taken from a recent study by Mayhew et al. (2012). All observers participated in one scanning session during which they performed a categorization task on Glass pattern stimuli (i.e. are the stimuli concentric or radial?). For each observer, we collected data from 7 or 8 event-related runs in each session. Each run comprised 129 trials (128 trials across conditions and one initial trial for balancing the history of the second trial) and two 9 s fixation periods (one in the beginning and one at the end of the run). Eight conditions (seven stimulus conditions and one fixation condition during which only the fixation square was displayed at the centre of the screen) with 16 trials per condition presented in each run. The stimulus conditions comprised Glass patterns of 0° ± 1:5° or 90° ± 1:5° spiral angle that were presented at 0, 25, 35, 50, 70, 85, and 100% signal levels. The order of trials was matched for history (1 trial back) such that each trial was equally likely to be preceded by any of the conditions. The order of the trials differed across runs and observers.

Each trial in the categorization experiment described above lasted 3 s. The categorization task involved three processes, i.e. (1) visual analysis (stimulus integration and processing), (2) perceptual judgement, and (3) motor response. Except for fixation trials, each trial started with 200 ms stimulus presentation followed by 1300 ms delay during which a white fixation square was displayed at the centre of the screen. The stimulus evoked both visual analysis and perceptual judgement with different process onsets, as indicated by the analysis of simultaneously collected EEG-fMRI signals. After this fixed delay, the fixation dot changed colour to either green or red. This change in fixation colour served as a cue for the motor response using one of two buttons. If the colour cue was green, observers indicated concentric vs. radial by pressing the left vs. right finger key, while if the colour was red, the opposite keys were used (e.g. concentric = right key). The fixation colour was changed back to white 300 ms before the next trial onset. The above procedure can dissociate the motor response process evoked by the cue for button press from the stimulus categories.

During the scanning sessions, EPI data (gradient echo-pulse sequences) were acquired from 24 slices (whole brain coverage, TR: 1500 ms, TE: 35 ms, flip-angle: 73°, 2.5 × 2.5 × 4 mm resolution). These parameters resulted in two MR volumes collected per trial. As we have 129 trials per run (*S* = 129), the number of fMRI measurements for each run is therefore 258 (*T* = 258). At this temporal resolution, the timing of visual analysis and perceptual judgement could not be separated in the context of the rapid event-related design used for the collection of fMRI data. A single process was therefore used to summarize these two processes. At the same time, we use another independent process to account for the button press. Thus, there exist two separate processes in each trial (*P* = 2).

Recall that two distinct but overlapping processes were evoked by the visual stimulus in each trial. Both the temporal characteristics and spatial locations of these two processes can be identified by an EEG-informed fMRI study. In this previous work we concentrated on two components that previous studies suggest reflect distinct processes. In particular, previous studies (Das et al., 2010, Johnson and Olshausen, 2003, Ohla et al., 2005, Tanskanen et al., 2008) showing differential responses to global forms at later rather than early latencies suggest that latencies around the first component (86–119 ms) relate to visual form integration, while latencies around the second component (229–249 ms) relate to perceptual classification judgements. Subsequently, the EEG amplitudes at these two time instances from all individual trials were used to construct two corresponding regressors in an EEG-informed GLM. This analysis identified a number of ROIs which were associated with the above processes.

We used a total of 320 independent data sets pooled across 10 participants, runs and ROIs to validate the spatio-temporal model presented in the Methods section. Moreover, we select four different ROIs involved in visual analysis and/or perceptual judgement: Middle Frontal Gyrus (MFG), Superior Frontal Gyrus (SFG), Primary Visual Cortex (V1), and Lateral Occipital Gyrus (LO). Note that MFG and SFG are two frontal ROIs whereas V1 and LO are two occipito-temporal ROIs. We would have *K* = 1 if a ROI were functionally homogeneous. When this assumption fails, the *K*-value should be greater than 1. Note that the threshold for ROI-determination was set to 0.05 (with cluster threshold correction) in the previous study. It is possible that our results may change when a different threshold value was used. Particularly, sub-ROI information could be altered if a smaller threshold value was used which would result in smaller ROIs. In this work, however, the ROIs to be analyzed in more detail by our method were fixed to those used in Mayhew et al. (2012).

### Description of synthetic data

**Fig. 3:**
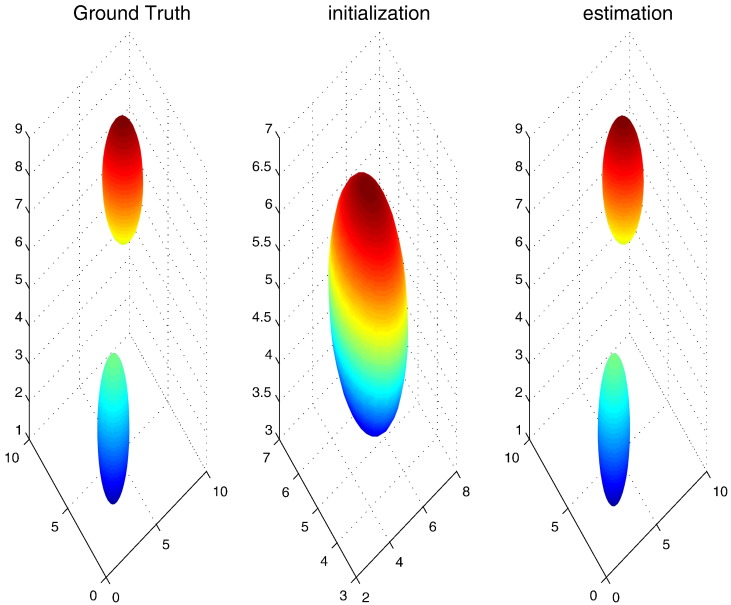
Numerical experiments with synthetic data: Plots of two 68%-isodensity ellipse *in the three-dimensional voxel space* representing the spatial distribution (or the region of influence) of two corresponding prototypes which constitute the model that generates our synthetic data: ground truth (left panel), an initialisation for gradient-based learning (middle panel), and reconstruction from the estimated spatial parameters (right panel). Very accurate estimates are obtained even with non-informative initialisation.

**Fig. 4:**
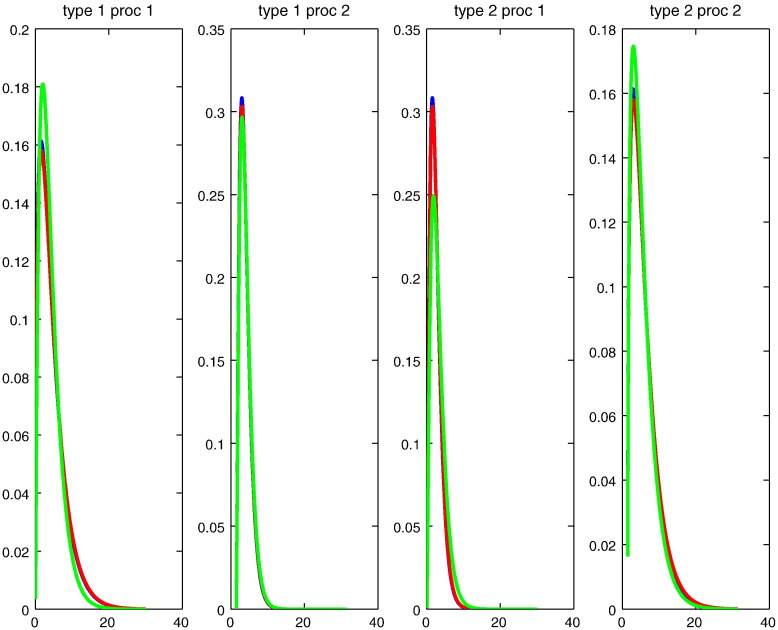
Numerical experiments with synthetic data (continued): Plots of haemodynamic response functions for every process defined in each of two prototypes that constitute the model that generates our synthetic data: blue: ground truth; green: an initialisation for parameter learning; red: reconstruction from the estimated temporal parameters.

**Fig. 5:**
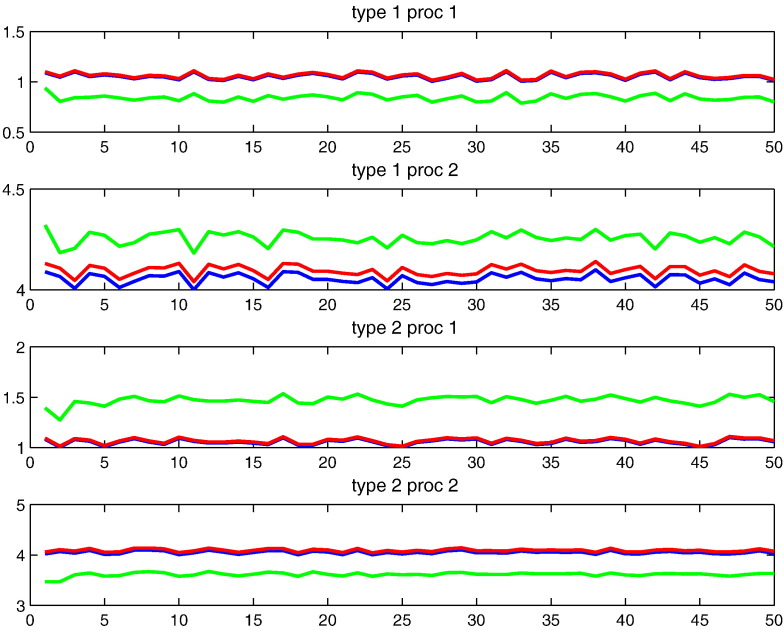
Numerical experiments with synthetic data (continued): Plots of response magnitude time series for every process defined in each of two prototypes that constitute the model that generates our synthetic data: blue: ground truth; green: an initialisation for parameter learning; red: reconstruction from the estimated temporal parameters.

As pointed out previously, an artificial fMRI volume that resembles real fMRI data is generated to assess how accurate the model parameters can be learned from data when compared to ground truth values. The size of that artificial volume is 10 × 10 × 10 (*V* = 1000), which is larger than the usual size of ROIs. Further, we consider that there exist two sources of neural activation. To account for this consideration, two prototypes of temporal models (*K* = 2) are set up. The Gaussian prior on the spatial distribution of their weights is displayed in the left panel in Fig. 3 in terms of 68% isodensity ellipses. The ground truth HRF for each of these two processes (*P* = 2) can be found in Fig. 4 (blue curves). Fig. 5 shows the temporal evolution of the corresponding response magnitudes (*S* = 50). Moreover, we set TR = 0.2 for generating the artificial data, which resulted in 250 fMRI measurements (*T* = 250).

### Results from synthetic data

As discussed in the previous section, model initialisation plays a crucial role in parameter estimation using gradient-based algorithm (see Methods section). To this end, a sophisticated initialisation procedure has been developed. For the above synthetic data, such procedure could produce the results of parameter estimation which are already quite accurate. On the other hand, it is also interesting to find out which kind of initialisation could lead to a failure in reasonable estimation of model parameter. For this purpose, we try a non-informative initialisation of spatial parameters as shown in the middle panel in Fig. 3. Further, we consider the initialisation of temporal parameter as a deviation from ground truth to some degree that varies from 1% to 20%. It turns out that for the deviation up to 10%, a good overall estimation of model parameter could still be achieved even with a non-informative initialisation of spatial priors (see Fig. 4, Fig. 5). For the deviation beyond this limit, a much better initialisation of spatial parameter is needed to obtain good results. For the example shown in Fig. 3, Fig. 4, Fig. 5, we have statistically consistent evidence showing *K* = 2 has significantly stronger model evidence than *K* = 1. To avoid determining burn-ins, we started with *K* = 2 and the MAP estimates of its model parameters. All models with different *K*-values were sampled for 1000 steps by a HMC sampler between two consecutive proposed moves (RJMCMC steps). These RJMCMC steps in turn are the backbone of Wang–Landau algorithm, which makes our algorithm a controlled RJMCMC algorithm.

### Results from fMRI data

The initialisation and learning algorithms described in the Methods section have been applied to estimate both spatial and temporal parameters for all 320 fMRI data sets. Some of them were discarded from further analysis as they contain a considerable amount of share motion artefacts.

To validate our method, we reconstruct fMRI signals *y*(*v*,*t*) as follows:

$$\hat{x}\left(v,t\right)=\sum_{k=0}^{K}\;p\left(k|y\left(v,t\right);{\hat{\Theta }}^{\mathrm{STM}}\right)\cdot x_{k}\left(t;{\hat{\Theta }}^{\mathrm{STM}}\right).$$

**Fig. 6:**
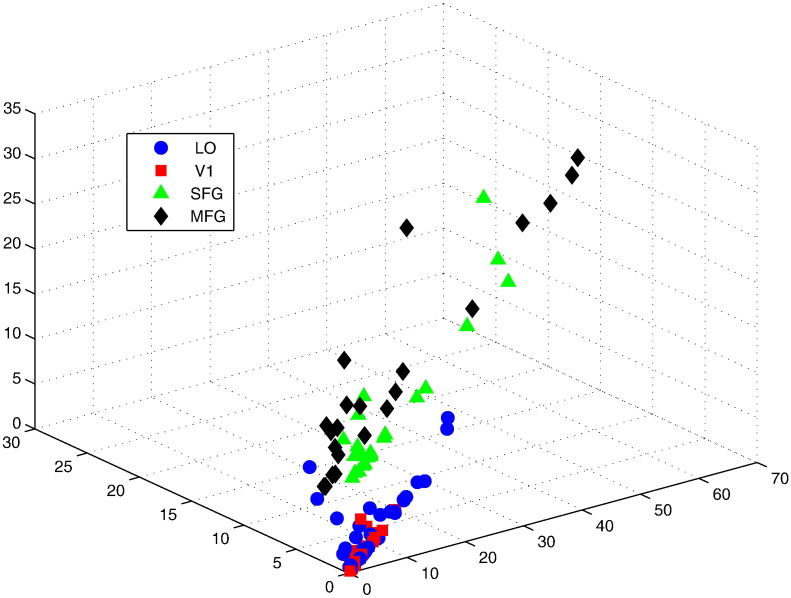
Reconstructed BOLD signals $\hat{x}\left(v,t\right)$ (red) and the fMRI measurements *y*(*v*,*t*) (blue) as function of fMRI volume index *t* for voxel *v*. We display the averaged signals and measurements, respectively, over all voxels in each of four different ROIs (LO, V1, MFG, and SFG).

Fig. 6 shows that signal reconstruction is very satisfactory in all ROIs. Averaged over all voxels in individual ROIs, one can hardly detect any difference between the real measurements and the reconstructed signals. This validation procedure is similar to so-called internal measures in standard cluster validation (Halkidi et al., 2001).

Attempts were made to further reduce model complexity by assuming that all stimuli in a particular condition have a fixed response magnitude for each fMRI data set. But this has greatly reduced the ability of our model to reconstruct fMRI signals. Consequently, this approach was not adopted.

One finding from the results is that the estimated HRF remains almost the same across runs, ROIs, and subjects whereas high variability is observed in the response magnitude and its temporal evolution. Also, the spatial distribution of prototypes shows some variability. The focus of our analysis is to answer how many prototypes are needed to adequately characterise fMRI data in single ROI.

#### Homogeneity vs. inhomogeneity within ROIs

Typically, single ROIs (or parcels) are often considered as anatomically and functionally homogeneous (Flandin et al., 2003). This implies that one prototype (together with the null one) is already sufficient to characterise a single ROI. To test this hypothesis, we first fixed the maximum number of prototypes in a single ROI to two, *K* = 2, and checked whether these two prototypes determined from data are largely the same. We also adopted a computationally expensive approach based on Bayesian model selection to determine how many prototypes are needed, in case one prototype was shown to be insufficient.

In particular, we studied:

1. to which degree the spatial distribution of two prototypes overlaps. For each data set, we computed a triple of symmetrized KL divergences, i.e. $\left({\mathrm{KL}}_{N_{0}N_{1}},{\mathrm{KL}}_{N_{0}N_{2}},{\mathrm{KL}}_{N_{1}N_{2}}\right)$, where $N_{1}$ and $N_{2}$ represent the Gaussian priors of two prototypes in the model while the isodensity ellipse of $N_{0}$ is used to approximate the 3D shape of ROIs. The results are displayed in Fig. 7;

**Fig. 7:**
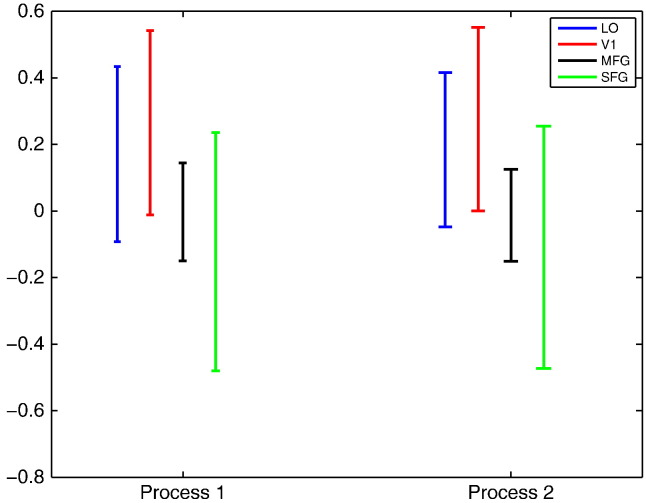
The *x* and *y* coordinates represent the measure of overlapping between the distribution of voxels and the distribution of prototypes 1 and 2, respectively, while the *z*-coordinate corresponds to the measure of overlap between prototypes 1 and 2. All data points of the same colour are from one particular ROI but across subjects and runs.
2. to which degree the temporal evolution of response magnitudes of the prototypes in the model is cross-correlated for a particular process, i.e. visual–perceptual process (referred as process 1) and decision-motor response process (referred as process 2). The computed correlation coefficients are displayed in Fig. 8 in terms of the mean and double standard deviation across runs and subjects.

**Fig. 8:**
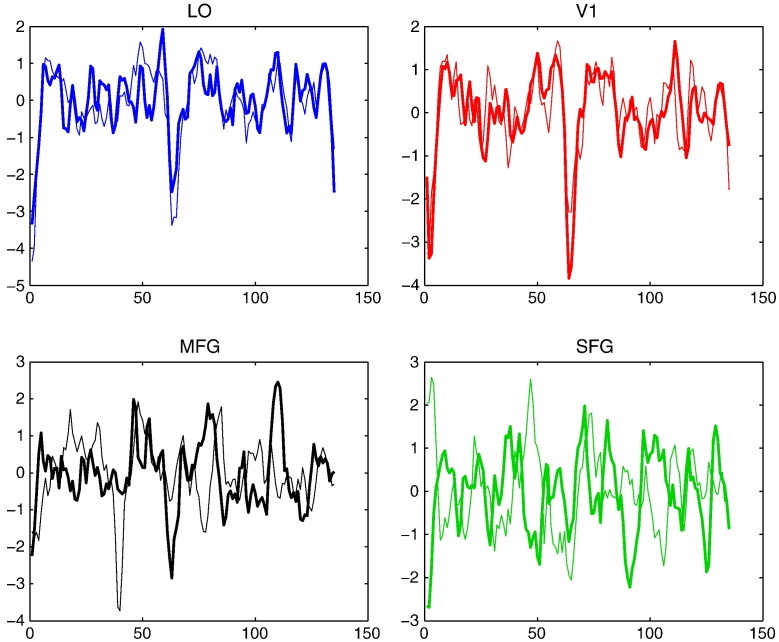
95% confidence intervals of the estimated cross correlation coefficients between the time evolution of response magnitudes of process 1 (visual analysis and perceptual judgement) and process 2 (motor-response) from prototypes 1 and 2.

Fig. 7 shows that the spatial distribution of two prototypes in the model is more overlapping for occipital (V1, LO) rather than frontal (SFG and MFG) ROIs. Moreover, Fig. 8 shows that two prototypical patterns of response magnitudes are positively correlated for V1 and LO whereas such evidence is not present for MFG and SFG.

**Fig. 9:**
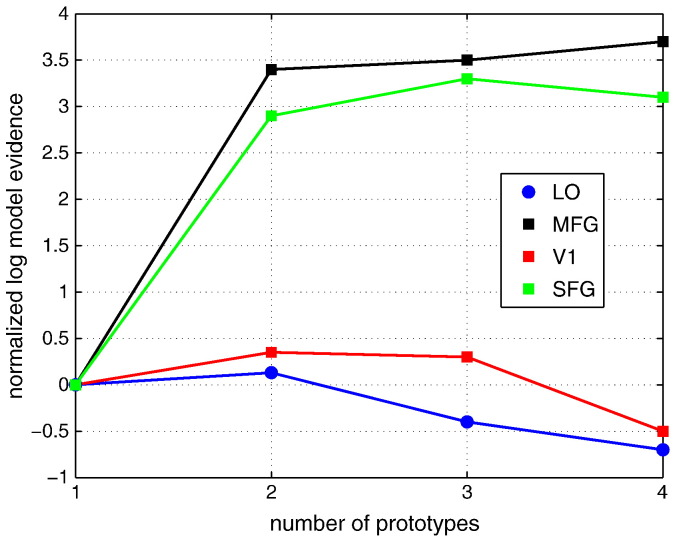
Log model evidence as function of the number of prototypes for 4 example data sets from different ROIs.

As discussed in the previous section, determining the number of necessary prototypes from fMRI data is computationally very expensive when model evidence is used. Therefore, approximation of model evidence, such as BIC or free energy is often used. In this work, the model evidence approach is tested with four example fMRI data sets. Each data set is derived from one of four ROIs that are considered in this work. Fig. 9 shows that two prototypes are clearly needed for SFG and MFG while for V1 and LO, a single prototype is probably sufficient.

**Fig. 10:**
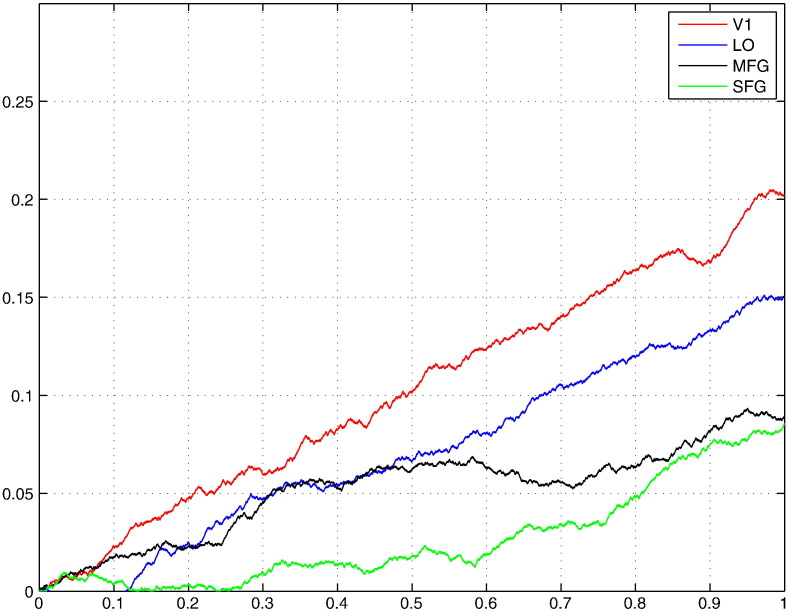
Time series of response magnitudes of process 1 (visual analysis and perceptual judgement) as function of stimulus index. The thick and thin curves in each panel show the time series from two different prototypes in individual ROIs. The same data sets were used as in Fig. 9 representing four different ROIs (LO, V1, MFG, and SFG).

Fig. 10 shows that the time series of response magnitudes are temporally correlated. The response magnitudes of prototypes 1 are positively cross-correlated with those of prototype 2 in two occipito-temporal ROIs (V1 and LO). This observation could support the assumption that these ROIs are functionally homogeneous. For two frontal ROIs (MFG and SFG), however, the negative cross-correlation is observed. This can be seen as a strong indication of functional inhomogeneity in MFG and SFG. These observations indirectly indicate that one needs more prototypes for modelling fMRI data in MFG and SFG.

To understand these results, it is important to differentiate between the cognitive processes represented in the considered ROIs and the model. This is shown in Table 1. The spatial homogeneity in occipitotemporal ROIs suggests that representation in these areas relates to a single process, namely Process 1 that focuses on visual analysis. Similarly, the inhomogeneity in frontal ROIs could be caused by overlapping representations related to Process 1, focusing on perceptual judgement, and Process 2 (motor-response process). Note that Process 1 in our model represents both visual analysis and perceptual judgement processes.

**Table 1:** Presence of different cognitive processes in the ROIs and their counterpart in the prototypical models.

Cognitive process	Frontal ROIs (MFG and SFG)	Occipitotemporal ROIs (V1 and LO)	Process in prototype
Visual analysis	No	Yes	Process 1
Perceptual judgement	Yes	No	Process 1
Motor response	Yes	No	Process 2

#### Understanding heterogeneity within ROIs

We have shown that our model selection mechanism (model evidence) clearly favoured more than a single prototypical HPM within frontal ROIs. In this section we will provide a more detailed analysis of this observation. One can think of several reasons why more than one prototype HPMs are needed to describe the representations in a ROI. For example, the local structure of hidden processes (e.g. HRF, response delays) can vary, requiring different local HPM prototypes. However, perhaps not surprisingly, we did not observe this level of variability within single ROIs. One can then ask: Where does the need for two prototypical HPMs come from? To answer this question, we study the series of response magnitudes for each process in each prototype.

Given a ROI, it is possible that one process is prominent in one local region, whereas another process is prominent in another local region of that ROI. Since both Process 1 and Process 2 are included in every HPM prototype, a direct hypothesis testing for the existence of a particular process within a local region, ‘governed’ by a particular HPM prototype, could be done by checking whether its response magnitudes are vanishingly small. However, this approach is not feasible because the fMRI data was normalized to zero-mean and unit variance. Consequently, the absolute value of response magnitudes estimated from fMRI data is interpretable only relatively with respect to other processes in the same HPM prototype.

Given that the HPM prototypes were found to be similar within individual ROIs, we hypothesise that if the need for more than one prototype arises, it is because at each time step one of the processes is more prominent in one prototype, whereas the other process is prominent in the other one. We next formulate a test for this hypothesis, considering the relative difference in response magnitudes between Process 1 and Process 2 in each of the two prototypes:

$$r_{s}^{k}=a_{1,s}^{k}-a_{2,s}^{k},$$

where *a*_*p*,*s*_^*k*^ is the response magnitude of process *p* in prototype HPM *k* to the *s*-th stimulus.

For each time step *s* (time point *t_s_*) we define a binary variable^3^

$$S_{s}=\mathrm{sign}\left(r_{s}^{1}\cdot r_{s}^{2}\right).$$

If at presentation of the *s*-th stimulus one of the processes is prominent in both prototypes (*r*_*s*_^1^ and *r*_*s*_^2^ will have the same sign), we get $S_{s}=1$ (indicating homogeneity of a single process within the whole ROI). On the other hand, if different processes are prominent in different regions of ROI, we will have $S_{s}=-1$. We concatenate such sequences across subjects and runs, resulting in a long sequence for each considered ROI.

**Fig. 11:**
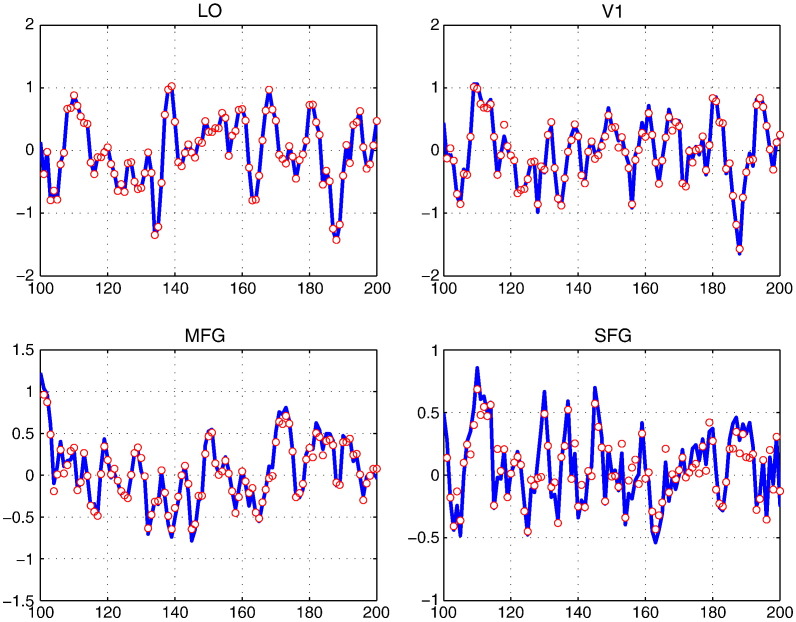
Plot of $l\left(s\right)$ against *t*_*s*_/*T* for two frontal ROIs (MFG and SFG) and two occipitotemporal ROIs (V1 and LO) where *T* is the total number of stimulus presentations across runs and subjects.

To visualise the difference among the four ROIs, in Fig. 11 we plot the cumulative sum of $S_{s}$, $l\left(s\right)=\sum_{i=1}^{s}\;S_{i}$, against *s* for each ROI. The curves $l\left(s\right)$ for two frontal ROIs (MFG and SFG) increase with *s* much slower than those for V1 and LO. Moreover, several considerably long sub-curves with negative slope are found for MFG and SFG. Fig. 11 shows that our hypothesis has more ground in the frontal regions than in the occipitotemporal ones. One possible interpretation of this finding is that in the occipitotemporal ROIs only Process 1 (focused on visual analysis) exists. Therefore, if two processes are used in prototypical HPMs in those ROIs, we should not obtain heterogeneity of response magnitudes indicated by negative $S_{s}$. Indeed, the $l\left(s\right)$ curves of occipitotemporal ROIs have much less negative contributions than those in frontal ROIs. This agrees with the fact that both Processes 1 and 2 are expected to be present in frontal ROIs, whereas we expect that only Process 1 exists in occipitotemporal ROIs (see Table 1).

In this section, we have presented a set of evidence supporting our hypothesis on functional homogeneity within individual ROIs. For each individual evidence, there may exist concerns about its statistical significance. Therefore, caution is needed to interpret those results. It is however remarkable that all evidence leads to the same conclusion.

## Discussion

We have presented a spatio-temporal model for the analysis of fMRI data in individual brain regions. In this model, spatio-temporal behaviour of fMRI time series is summarized by a small number of prototypical temporal patterns. In our setting a prototypical temporal pattern is a distribution of possible BOLD signals within a single voxel defined through a Hidden Process Model (HPM). Each temporal prototype comes with a spatial prior over the voxel space which determines its “region of influence” over voxels in its vicinity; mixture-of-experts. We have also presented a tailored optimization algorithm that is used to determine the spatial prior of every prototype, as well as the HRF of every process in the prototypes and the corresponding time series of response amplitudes. This computationally efficient MAP algorithm is further extended to a MCMC algorithm that can determine the number of prototypes in a Bayesian model selection setting. We evaluated our principled framework in a controlled experimental setting on the task of identifying prototypical spatio-temporal patterns of real neural activation evoked by visual stimuli within several pre-determined ROIs. As expected, the within-ROI variation of neural activations inferred from the model differs substantially between frontal and occipital ROIs.

In this work, we have adopted a HPM approach to model single-voxel fMRI time series. The essence of this approach is to treat the contribution of overlapping cognitive processes to the observed data separately. For the cognitive experiments from which our data were generated, it is of interest to separate the process related to stimulus analysis and perceptual judgement from the process related to the motor response. Note that in our experimental setting the onsets of the two processes were separated by about 1.5 s. However, the process evoked by the stimulus is a lumped process comprising of visual stimulus analysis and perceptual judgement. Separating and understanding such processes that occur very close in time (at the temporal scale of ms) is of key importance in cognitive neuroscience, which might be more interesting. To this end, two approaches can be adopted:

1. The decision whether one lumped process or two separate processes should be used in the temporal model can be formulated as a model selection problem. For example, following Hutchinson et al. (2009) the two models (one model with a single lumped ‘visual/perceptual’ process, the other model considering visual analysis and perceptual judgement processes as two separate modelling entities) can be compared in a data driven manner through cross-validation.
2. Following the methodology introduced in this study, one can impose within a single model two separate visual and perceptual parameterized processes and then learn the global model using fMRI data. Using the fitted model one can then compare the inferred individual processes. This approach is not only computationally more efficient, but crucially, it also allows for further model based analysis, e.g. analysis of the difference in spatial variation of these two processes.

In this study, we have adopted the second approach to disentangle the perceptual judgement processes from the motor response processes in frontal ROIs (MFG and SFG). It was found that there ought to exist two different processes in these ROIs. According to our model specification, one of them (Process 1) is activated shortly after the stimulus presentation and another one (Process 2) is activated after participants were asked to perform motor response. This finding was obtained by analyzing the series of response magnitudes estimated for Processes 1 and 2 in each of two prototypes. A plausible explanation of the observed evolution of response amplitudes is that within frontal ROIs, processes 1 and 2 have diverse dynamic localizations — which process is prominent in which local sub-region of a given ROI changes over time. This makes the frontal ROIs “functionally inhomogeneous”. Also, we found that V1 was the most homogeneous ROI. This agrees with the fact that V1 is involved primarily in visual analysis, whereas LO could be involved not only in visual analysis but also in perceptual judgement through feedback from frontal regions.

In model-based fMRI analysis, the inference of temporal fMRI models can be rather complex as the temporal resolution of fMRI data is typically low. Therefore, temporal constraints are usually imposed. The strongest constraint one can find in the literature is as follows: 1) the HRF is fixed and known; and 2) response magnitudes are unknown but constant in time. As mentioned in the Introduction section, it is now generally accepted that the HRF needs to be learned from fMRI data sets (Aguirre et al., 1998). However, *it is still reasonable to assume that the HRF is fixed within a single session* (Donnet et al., 2006) *and across neighbouring voxels* (Flandin et al., 2003). In this work, we take this view of HRF variability, but allow for HRF to differ between the overlapping cognitive processes. However, the constraint of constant (within session) response magnitude is still commonly used (e.g. Hutchinson et al., 2009). This view is questioned in Donnet et al. (2006) as the assumption of constant response levels may not hold for (rapid) event-related neuroimaging experiments of the kind used in our work. Two solutions to this problem have been proposed in Boynton et al. (2006), Donnet et al. (2006), and Ciuciu et al. (2010): 1) the response levels are considered as i.i.d. Gaussian distributed random variables. The means and variances are estimated from data; and 2) the response levels can still be considered as constant for all stimuli of the same type, but are allowed to vary across the stimulus types. The first approach is computationally very expensive while the second one would not work for our fMRI data. In our work, all stimuli are of the same type but vary in the signal-to-noise ratio of Glass patterns. Therefore, we estimate the response levels for each stimulus. It is worth mentioning that in our approach the estimation of HRF may interfere with that of the response levels — we use Gamma function as the parametric form of HRF and hence the height of HRF varies with its shape and scale parameter settings. This implies that the response level and HRF form should be jointly considered in order to properly interpret our results presented in the previous section. In a fully Bayesian treatment the posterior distribution would be characterised by one-dimensional equi-probability structures in the HRF height vs. response amplitude plane. However, it is interesting that despite no explicit constraints on HRF parameters and response amplitudes, under the MAP estimation adopted in this study, the HRF heights were almost the same across all considered ROIs.

Another distinct aspect of our model is that a probabilistic mixture-of-experts approach is adopted to jointly take into account several possible temporal patterns. This idea can also be implemented in a GLM setting as in Gershman et al. (2011). Both approaches are based on the so-called superposition principle, albeit in two different ways. In our model, the superposition is mathematically formulated as a mixture in model space whereas the approach adopted in Gershman et al. (2011) is formulated in terms of a mixture in signal space (also called mixing). For the mixture in model space, the determination of the number of mixture components has been extensively studied in the literature and a Bayesian approach to this problem has been built on a sound theoretical foundation. Thus, our approach can be more promising than mixing in tackling the problem of model selection in fMRI analysis. Of course, this is not limited only to activation detection. Similarly, the mixture-based approach would also allow us to integrate out model uncertainty in a principled manner as in Hutchinson et al. (2009). More importantly, the mixing approach could make the disentanglement of overlapping processes impossible because there is an identifiability problem between the prototypes and processes. An alternative to our approach is so-called Hierarchical Clustering as adopted by Hutchinson et al. (2009), which is computationally more time-consuming than ours.

The spatial aspect of our model is mainly reflected in the way the mixture coefficients are spatially regularized. Loosely speaking, this is about modelling spatial “spheres of influence” of our HPM prototypes (spatial fields). Essentially, there are two classes of approaches: 1) the random field approach and 2) the basis function approach. The difference in these two approaches has already been highlighted in the Introduction section. Both our approach and the one presented in Flandin and Penny (2007) are two examples of the 2nd class, but differ subtly. This is because a set of fixed basis functions, i.e. wavelet functions, is used in Flandin and Penny (2007), whereas we estimate those basis functions from the data. The basis functions have a canonical form, namely a three-dimensional Gaussian. The advantage of our approach is that it would allow us to naturally incorporate prior knowledge. Similar problems were encountered in the semi-parametric approach to HRF modelling (Woolrich et al., 2004a), in which non-sensible HRFs could be produced. In Gershman et al. (2011), three-dimensional Gaussians with isotropic covariance matrices are used, which would introduce severe restriction on the shape of “region of influence”. Thus, full covariance matrices are used for the prototypes in our model. An extension to using more complicated spatial basis functions, as those proposed in Friman et al. (2003), is straightforward.

Most of the previous fMRI studies have focused on modelling the temporal dynamics of BOLD signals at short time scales while the inter-sessional variability is often considered as a random effect (van Gerven et al., 2008). However, it is of great interest to model this large-scale variability of haemodynamic responses in a more general setting. This would find applications in various areas. Two examples: (1) In cognitive science, it is known that learning changes BOLD signal responses to cognitive tasks (Duff et al., 2007, Mayhew et al., 2012). To understand the neural mechanisms that support improvements due to learning, those changes need to be interpreted consistently and specific hypothesis needs to be tested. (2) In clinical applications, it would be very helpful to select best treatment for individual psychiatric or neurological disorder patients if the brain response to treatment could be tracked and predicted (Guo et al., 2008). In both cases, it is advantageous to develop fMRI models that can account for the temporal correlations between BOLD signal responses across several sessions in a sequence. One challenging problem is how to deal with the increased computational burden. One solution is to select one or several representative voxels for each ROI, as it is often the case for group analysis or meta analysis of fMRI data. However, this may not provide a sufficient characterisation of BOLD signals across a single ROI. In contrast, the spatio-temporal prototypes derived from our fMRI model represent a sparse but yet sufficient characterisation of fMRI data within single ROIs.

## Footnotes

1 Including possible HRF shapes for all evoked neural processes.

2 In Neural Gas (as an on-line training method) the order in which training inputs are applied can have influence on the final clustering solution. Indeed as pointed out in Qin and Suganthan (2004): *… the initialisation problem is implicitly converted to the input sequence ordering issue for the sequential learning method*.

3 In case *a*_1,*s*_^*k*^ = *a*_2,*s*_^*k*^, we put *r*_*s*_^*k*^ = − 1.
